# PARTIAL: study protocol for a clinical and cost-effectiveness of complex PARTIAL vs radical nephrectomy for clinically localised renal cell carcinoma randomised trial

**DOI:** 10.1186/s13063-026-09624-4

**Published:** 2026-03-20

**Authors:** Diana Johnson, Ruth Thomas, Seonaidh Cotton, Rumana Newlands, David Cooper, Sharon McCann, Luke Vale, Katie Gillies, Axel Bex, Ben Challacombe, Jemma Falloon, Graeme MacLennan, Krishna Narahari, David Nicol, Neil Sheerin, Grant D. Stewart, Maxine Tran, Rakesh Heer, Naeem Soomro

**Affiliations:** 1 Centre for Healthcare Randomised Trials, Aberdeen Centre for Evaluation, University of Aberdeen, Aberdeen, UK; 2 Aberdeen Centre for Evaluation, University of Aberdeen, Aberdeen, UK; 3 London School of Hygiene & Tropical Medicine, London, UK; 4 Royal Free London NHS Foundation Trust, London, UK; 5 Guy’s and St Thomas’ NHS Foundation Trust, London, UK; 6 Kings College London, London, UK; 7 Patient and Public Involvement Member Based in England, London, UK; 8 Cardiff University, Cardiff, UK; 9 Cardiff and Vale University Health Board, Cardiff, UK; 10 The Royal Marsden NHS Foundation Trust, London, UK; 11 University of Newcastle upon Tyne, Newcastle, UK; 12 Department of Surgery, University of Cambridge, Cambridge, UK; 13 University College London, London, UK; 14 Imperial College London, London, UK; 15 The Newcastle Upon Tyne Hospitals NHS Foundation Trust, Newcastle, UK

**Keywords:** Kidney cancer, Nephron-sparing surgery, Radical nephrectomy, Renal cell carcinoma, Renal function

## Abstract

**Background:**

Localised renal cell carcinoma is treated with radical nephrectomy (RN) or partial nephrectomy (PN). Nephron-sparing PN increases preservation of renal function, reducing incidence of end stage renal failure and associated cardiovascular events. In patients with exophytic T1a (≤ 4 cm) tumours and normal contralateral kidney, PN is standard of care. In patients with T1b (> 4–7 cm) or endophytic T1a tumours and normal contralateral kidney, the benefits of PN over RN are less clear as there are increased surgical complications and more tissue may be excised reducing the preservation of renal function. There are no high-quality studies to address if PN is superior to RN in these more complex cases.

**Methods:**

PARTIAL is a pragmatic randomised controlled parallel group unblinded superiority trial with embedded internal pilot and economic and process evaluation. A total of 420 participants will be recruited in UK NHS centres with expertise in minimally invasive nephrectomy techniques. Eligible consenting adults with a single T1 renal cell carcinoma, normal contralateral kidney and equipoise within the multidisciplinary team confirming suitability to receive both interventions by minimally invasive approaches are randomised 1:1 to PN or RN. Patients with metastatic disease, existing chronic kidney disease, solitary functioning kidney, congenital renal abnormality, inherited kidney cancer syndrome, who lack capacity to consent or are pregnant or breast feeding are excluded. Primary outcomes are gains in preservation of renal function at 2 years and surgical complications over the peri-operative period. Secondary outcomes are quality of life and recovery, cost and cost-effectiveness, rates of positive surgical margin, recurrence and cardiovascular events, overall survival, progression to chronic kidney disease and end stage renal failure, operative conversion and patient acceptability. Participants are followed up for 2 years with outcomes collected from medical records and participant questionnaires.

**Discussion:**

PARTIAL will determine if gains from PN are superior to RN and offset the potential harms and costs in complex T1 renal tumours suitable for either approach. If PN is not found to provide clinically significant gains and excess complications are confirmed, then a practice-changing case for RN as standard of care could be made.

**Trial registration:**

ISRCTN 11293415. Registered prospectively on 19 January 2023.

**Supplementary Information:**

The online version contains supplementary material available at 10.1186/s13063-026-09624-4.

## Administrative information

Note: the numbers in curly brackets in this protocol refer to SPIRIT checklist item numbers. The order of the items has been modified to group similar items (see http://www.equator-network.org/reporting-guidelines/spirit-2013-statement-defining-standard-protocol-items-for-clinical-trials/).

**Table:** 

Title {1}	PARTIAL: study protocol for a clinical and cost effectiveness of complex PARTIAL vs radical nephrectomy for clinically localised renal cell carcinoma randomised trial.
Trial registration {2a and 2b}	ISRCTN 11293415. Registered prospectively on 19 January 2023.
Protocol version {3}	Version 3, 01 August 2024
Funding {4}	National Institute for Health Research (NIHR) Health Technology Assessment (HTA) Programme (NIHR133561).
Author details {5a}	^1^Centre for Healthcare Randomised Trials, Aberdeen Centre for Evaluation, University of Aberdeen, Aberdeen, UK. ^2^Aberdeen Centre for Evaluation, University of Aberdeen, Aberdeen, UK. ^3^London School of Hygiene & Tropical Medicine, London, UK. ^4^Royal Free London NHS Foundation Trust, London, UK. ^5^Guy’s and St Thomas’ NHS Foundation Trust, London, UK. ^6^Kings College London, London, UK. ^7^Patient and Public Involvement member based in England, UK. ^8^Cardiff University, Cardiff, UK. ^9^Cardiff and Vale University Health Board, Cardiff, UK. ^10^The Royal Marsden NHS Foundation Trust, London, UK. ^11^University of Newcastle upon Tyne, Newcastle, UK. ^12^Department of Surgery, University of Cambridge, Cambridge, UK. ^13^University College London, London, UK. ^14^Imperial College London, London, UK. ^15^The Newcastle Upon Tyne Hospitals NHS Foundation Trust, Newcastle, UK.
Name and contact information for the trial sponsor {5b}	The Newcastle upon Tyne Hospitals NHS Foundation Trust Level 1, Regent Point, Regent Farm Road, Gosforth, Newcastle upon Tyne, NE3 3HD tnu-tr.sponsormanagement@nhs.net
Role of sponsor {5c}	Sponsor has no role in the study design; the collection, management, analysis or interpretation of data; or the writing or submission of reports for publication.

## Introduction

### Background and rationale {6a}

Widespread use of imaging investigations has led to a major increase in incidental diagnoses of early small kidney cancers. Incidence has doubled over the last 20 years and is projected to increase further [1]. Kidney cancer is the 7th most common cancer in the UK with over 13,000 new cases per year [2]. Renal cell carcinoma (RCC), the commonest type of kidney cancer, was historically managed by radical nephrectomy (RN) to remove the whole kidney. Most of these patients managed without significant problems with their remaining kidney [3], which partly compensates for the lost function. In those patients with pre-existing chronic kidney disease (CKD) or a solitary kidney, nephron-sparing partial nephrectomy (PN) became the preferred surgical treatment option to remove only the portion of the kidney containing the cancer. PN is technically more challenging and associated with increased surgical complications, positive surgical margins and recurrence rates. However, in situations of renal compromise, these increased risks were offset by maintained renal function that in turn potentially reduced cardiovascular events and helped avoid CKD and end stage kidney disease (ESKD) [4].

Experience of PN has grown and its application has expanded to include small peripheral tumours (≤ 4 cm, T1a) in patients with a normal contralateral kidney. With surgical advances afforded by robotic surgery [5], European Association of Urology and major US guidelines recommend that surgeons tackle intermediate sized tumours (4–7 cm, T1b) and/or higher complexity surgeries involving central (hilar) T1a tumours with PN in those patients with normal contralateral kidney [4, 6, 7]. However, PN to treat larger and/or more complex tumours can be associated with (i) a greater loss of renal mass and renal function and (ii) higher risks of significant perioperative complication and positive surgical margins [8].

Currently, there are no ongoing RCTs of PN vs RN for surgical treatment of smaller renal tumours. Previous evidence syntheses have relied predominantly on retrospective cohort data [9, 10]. A Cochrane review identified the only randomised controlled trial (RCT) comparing PN and RN for small RCCs (< 5 cm) [11]. This RCT did not meet its pre-planned accrual targets and the technical approaches to PN were still being refined. A 21% crossover occurred, mainly PN to RN due to perceived technical challenges, affecting the intention-to-treat analysis [12]. In terms of overall survival, non-inferiority of PN was not demonstrated [13]. Given the absence of high-quality evidence, the authors of the Cochrane review recommended a methodologically rigorous study to assess the potential benefits and harms of PN, whilst accounting for baseline renal function, tumour size and patient age [11]. Technical advances with minimally invasive robotic PN have rapidly progressed and the clinical climate is now receptive to the conduct of an RCT. More recently, < 5% of procedures were reported to convert from PN to RN [14–16].

Following both PN and RN, renal function initially falls but compensations can occur with within the first 9 months after surgery and stabilises beyond the first 12–18 months [3, 17]. In Van Poppel et al., mean estimated glomerular filtration rate (eGFR) stabilised at 12 months at 52.7 ml/min/1.72m^2^ with RN and 66.8 ml/min/1.72m^2^ with PN [12]. Reductions of renal function to these levels are associated with a 5-year risk of (i) ESKD approaching 1% in both groups [3] and (ii) cardiovascular events of 9.9% and 15.6%, for PN and RN respectively [18, 19]. Although not reproduced in the Van Poppel study, these risks of cardiovascular events are consistent with the 40% increase shown by comparing eGFR > 60 vs GFR = 45–59 ml/min/1.73m^2^ in a longitudinal cohort study that is a seminal paper in the nephology field [20].

Most complications for both PN and RN occur acutely in the peri-operative period, such as bleeding and urine leaks. The main determinants of perioperative complications relate to the anatomy of the tumour—captured and categorised by a number of renal morphometric scoring systems, such as the Preoperative Aspects and Dimensions Used for Anatomic (PADUA) classification and Radius Exophytic/endophytic Nearness Anterior/posterior Location (RENAL) nephrometry score [21, 22]. The main variables determining complexity and risk of complication are size, higher-risk endophytic or favourable exophytic growth pattern, and higher-risk hilar locations next to main feeding vessels versus peripheral locations [8, 23]. Work from our group shows that depending on surgical complexity, moderate to severe complications (Clavien-Dindo 3 or higher) are seen in 4–11% of PN compared with just 2–3% in RN treated by robotic approaches [22]. Acceptable thresholds of complication are not well described, and most studies fail to provide information about the cumulative severity of complications, or inform only on the most severe event, ignoring events of lesser severity.

To define the key parameters for our trial, we undertook detailed surveys and focus groups with key stakeholders (patients, urologists and nephrologists). Patients with lived experience of RN or PN (*n* = 24) agreed that a clinical trial is urgently required to determine which treatment is superior in terms of relative benefits and harms for intermediate sized tumours. They shared that main drivers in decision-making were an intuitive and strong desire to maintain kidney function and avoidance of harm by prioritising being cured of cancer and returning to normal activities as soon as possible. The main drivers for urological surgeons (*n* = 110) considering PN vs RN are potential harms (related to the anatomy of the tumour). Otherwise, there was a strong conviction to save renal function where possible. In cases of T1b tumours (with the solitary exception of hilar endophytic T1b cases) and endophytic hilar T1a tumours, there was equipoise from urological surgeons regarding the best approach. Nephrologists (*n* = 32) chose an eGFR difference of at least 10 ml/min/1.72m^2^ between PN and RN as being a minimal clinically important difference and, triangulating these findings with those from the urologists and patients, informed the design of our trial and its co-primary outcomes.

## Objectives {7}

The aim of this trial is to determine the trade-off between potential benefits, harms and cost between partial nephrectomy compared with radical nephrectomy, for intermediate sized and selected small complex kidney tumours, primarily focusing on renal function and surgical complications over 24 months.

The hypothesis being tested:

Partial nephrectomy for intermediate sized 4–7 cm (T1b) and small < 4 cm (T1a) endophytic (deep-seated) tumours results in better renal function by at least 10 ml/min/1.72m^2^ compared to radical nephrectomy in patients with a normal contralateral kidney.

## Trial design {8}

PARTIAL is a pragmatic patient-randomised controlled, parallel group superiority trial, with an embedded economic evaluation comparing PN and RN for patients with suspected or confirmed renal cell carcinoma (RCC). Patients are approached to participate following informed decision-making and electing for a surgical approach. Patients are allocated to PN or RN in a 1:1 ratio. Trial treatments follow routine clinical management protocols.

The trial includes an internal pilot phase with an embedded mixed-methods process evaluation which aims to identify significant challenges relating to design or conduct that can be addressed and modified before progression to full trial.

## Methods: participants, interventions and outcomes

### Study setting {9}

Participants are recruited from NHS secondary care medium- and high-volume sites in the UK. A list of current trial sites can be obtained from the PARTIAL trial website [24].

### Eligibility criteria {10}

Participating sites are required to have surgeons and surgical teams competent in the delivery of robotic PNs and minimally invasive RNs. Surgeons have completed, as the primary surgeon, more than 30 robotic PNs and 50 laparoscopic or robotic RNs and, at the discretion of the chief investigators, more than 10 robotic PNs per year for the last 24 months. Median length-of-stay and surgical complication rates have to be within acceptable parameters. Individual surgeon data will act as surrogate measures for the entire surgical team.

The inclusion criteria for participants are:

- Adults ≥ 18 years,
- Newly diagnosed clinically localised renal cancer (suspected on cross-sectional imaging or histologically confirmed),
- Local multi-disciplinary review identifying those cases thought suitable for both minimally invasive RN or PN (for minimally invasive, we mean laparoscopic or robotic surgery; cases where open surgery is planned are not eligible),
- Cross-sectional imaging showing a single tumour, stage T1 (up to 7 cm), where there is equipoise in the multidisciplinary team (MDT) and willingness to recruit into the trial,
- On imaging, evidence of a radiologically normal contralateral kidney,
- Patients that have been fully counselled of all the available treatment options (including non-surgical approaches, where appropriate),
- Able and willing to give informed consent to participate and to participate in trial procedures.

The exclusion criteria for participants are:

- Solitary functioning kidney,
- Metastatic disease,
- Existing CKD (> stage 3b; eGFR < 45),
- Medically unfit for surgery,
- Congenital renal abnormality which includes fusion, assent and malrotation,
- Suspected or confirmed inherited kidney cancer syndrome,
- Current pregnancy or breast feeding,
- People without capacity.

Participant eligibility is confirmed by the principal investigator or a medically qualified delegate.

Participants must be eligible for the randomised trial to be eligible for the process evaluation.

### Who will take informed consent? {26a}

Eligible patients are given or sent the Participant Information Leaflet and supplement (the Decision-Making Tool). They have the opportunity to read the Participant Information Leaflet and supplement (the Decision-Making Tool) and discuss the trial with a member of the local trial team before deciding if they wish to take part. Potential participants are given enough time, and as long as they want, to accept or decline involvement. Consent is discussed face-to-face, virtually or by telephone and followed by hardcopy or electronic written informed consent by Good Clinical Practice (GCP) trained members of the local trial team and in accordance with GCP guidelines. Hardcopy written consent may be completed at home by the participant and returned in the post. The member of the local trial team who discussed consent with the participant countersigns the consent form.

The process evaluation component is optional for sites and patients. Data collection includes audio-recording the consultations when site staff (the recruiter) discusses the trial with the patient (to explore opportunities to improve the informed consent discussion), and interviews with patients and site staff (to identify opportunities to improve trial processes). Patients and the recruiter have the opportunity to read a Participant Information Leaflet. A one-off written consent is sought from the recruiter to audio-record the consultation, which covers all audio-recordings made by the recruiter for the trial. The member of the trial office who discussed consent to the audio-recording countersign the consent form. The local trial team seeks verbal consent from the patient to audio-record the consultation. The process evaluation researcher seeks verbal consent from patients and site staff for interviews. Verbal consent is audio-recorded. If verbal consent is declined, the audio-recording is stopped and the file deleted.

### Additional consent provisions for collection and use of participant data and biological specimens {26b}

Participants can opt to be contacted about participating in future long-term follow-up and relevant ethically approved research. Biological specimens are not collected in PARTIAL.

## Interventions

### Explanation for the choice of comparators {6b}

The two surgical technologies being compared are minimally invasive partial nephrectomy (PN) versus minimally invasive radical nephrectomy (RN).

Minimally invasive approaches are laparoscopic or robotic. The robotic approach is a minimally invasive laparoscopic approach assisted by robotic technology. Open approaches are significantly more morbid than minimally invasive approaches and usually reserved for very large or anatomically complex tumours, which are outside the inclusion criteria of our trial.

In the UK, in 2019, for patients with kidney cancers undergoing surgical treatment, there were 3130 RN (73% laparoscopic, 5% robotic, 19% open, 3% n/a) and 1562 PN (76% robotic, 9% laparoscopic 14% open, 1% n/a) as reported in the BAUS Nephrectomy Audit [25] capturing 79% of national activity. In England, in 2019–2023, for patients with kidney cancers undergoing surgical treatment, there were 17,216 RN (57% laparoscopic, 19% robotic, 23% open) and 6665 PN (82% robotic, 5% laparoscopic, 12% open) as reported in the National Kidney Cancer Audit State of the Nation Report 2024 [26]. There is evidence that both laparoscopic approaches, with or without the robot, are broadly similar in outcomes [27].

In the UK, robot access is increasing and the differential costs between laparoscopic and robotic costs are closing, meaning that an increasing proportion of robotic RN and PN is performed [28]. Therefore, to provide a generalisable approach to this pragmatic trial, we include both laparascopic and robotic minimally invasive approaches and exclude those for which open surgery is recommended.

### Intervention description {11a}

Minimally invasive approaches (laparoscopic or robotic) of RN and PN are undertaken in accordance with current NHS standard of care, in patients with diagnosed or suspected localised kidney cancer. RN and PN are performed (or supervised) by delegated surgeons who meet the minimum surgical experience criteria (described in eligibility criteria).

### Criteria for discontinuing or modifying allocated interventions {11b}

Where deemed clinically necessary, the intervention may be converted to an open procedure, or to a treatment different to that allocated at randomisation, or may not proceed at all. Participants who do not receive their allocated treatment are not considered withdrawals and will be followed up for all trial outcomes unless they request otherwise.

Participants are free to withdraw from the trial at any timepoint and can withdraw from participating in the intervention and/or other aspects of the trial. All changes in status, except for the complete withdrawal of consent, mean that the participant is still followed up for all trial outcomes wherever possible.

### Strategies to improve adherence to interventions {11c}

The inclusion criteria for the trial requires the multi-disciplinary team to confirm the participant is suitable to receive both interventions and for the participant to have been counselled of all available treatment options (including non-surgical approaches). The site team records in the case report form if the participant received the procedure allocated by randomisation. Any change from the allocated procedure is recorded with the reason stated.

### Relevant concomitant care permitted or prohibited during the trial {11d}

Participants will be permitted to take part in other non-interventional studies (e.g. questionnaire studies, studies collecting blood/tissue samples or studies investigating aspects of robotic surgery). Patients will be eligible for inclusion in PARTIAL if they are in the long-term follow-up phase of any other interventional trial. People enrolled in the active intervention phase of another interventional trial should be excluded from PARTIAL, except for trials of neo-adjuvant or adjuvant therapy. Participants will receive all appropriate concomitant care during their time in the study; no concomitant care is prohibited.

### Provisions for post-trial care {30}

The interventions are standard of care procedures. The surgery is a single procedure for the participant. At the end of the trial, participants will continue to be monitored as per standard NHS care. If a participant is harmed by taking part in the trial, they have the right to pursue a complaint and seek compensation through the research sponsor of the trial. If a participant is harmed due to negligence, they have grounds for legal action as a patient of the NHS.

### Outcomes {12}

Co-primary outcome measures:

(i) Gains in preserving renal function as measured by eGFR (using the Chronic Kidney Disease Epidemiology Collaboration (CKD-EPI) 2009 formula without the race coefficient as recommended by the National institute for Health and Care Excellence, the UK Kidney Association and European Federation of Clinical Chemistry and Laboratory Medicine [29–31]). The adjusted mean difference between the eGFR score in the PN and RN groups will be analysed to determine if PN results in better maintenance of eGFR compared with RN. This co-primary outcome timepoint is at 2 years post-randomisation, and it will be possible to present effect sizes at all follow-up points using a repeated measures model.
(ii) Harms captured by Comprehensive Complication Index (CCI). The CCI is validated in renal surgery [32, 33] and based on the Clavien-Dindo classification (reporting on the most severe event) and is calculated as the sum of all surgical complications that are weighted for their severity [34]. Ideally, the CCI will be analysed as an adjusted mean difference between the PN and RN groups, but the distribution or heteroscedasticity may require a different analysis. This co-primary outcome timepoint is reported at 3 months post-intervention (i.e., collecting events over the peri-operative period after skin closure for the index procedure to three months after surgery).

Secondary outcome measures:

- Health-related quality of life (HRQoL) captured by the European Organisation for Research and Treatment of Cancer Quality of Life Questionnaire Core 30 (EORTC QLQ-C30) [35] and 36-Item Short Form Survey Instrument (SF-36v2®, Acute version – 1 week recall) [36] participant questionnaires (PQs). The adjusted mean difference in the outcomes between the PN and RN groups will be measured over 2 years post-randomisation, and effect sizes can be presented at all follow-up points using a repeated measures model.
- Cost and cost-effectiveness captured by quality adjusted life years (QALYs) based on the Short Form 6 Dimension (SF-6D) algorithm and care pathway costs (including complications). The adjusted mean difference between the PN and RN groups will be analysed as incremental cost in UK pounds sterling (£) per QALY gained and reported as additional £ per QALY gained. Estimates will be based on data collected during the 2 years post-randomisation and presented at 2 years post-randomisation.
- Quality of recovery captured by the 15-Item quality of recovery (QoR-15) [37] PQ (including length of stay) to assess more subtle harms in the peri-operative timeframe from the patient perspective. The adjusted mean difference between the PN and RN groups will be measured over 3 months post-intervention, and effect sizes can be presented at all follow-up points using a repeated measures model.
- Residual disease captured by rates of positive surgical margin and need for retreatment/surgical revision. The difference between the PN and RN group will be indicated by an odds ratio and presented as the percentage in each surgical margin category. Positive surgical margin will be measured at the point of discharge and retreatment/surgical revision over 2 years post-randomisation.
- Recurrence-free and overall survival (including local recurrence) captured by cancer-specific survival and all-cause mortality. The difference between the PN and RN group will be indicated by a hazard ratio. The number of recurrences and time to recurrence will be presented for both groups. The summary statistics for the time to recurrence will be the median and quartiles. The outcome will be measured over 2 years post-randomisation.
- Rates of cardiovascular events captured by non-fatal heart attack, non-fatal stroke and cardiovascular death to assess the correlation between renal function, cardiovascular events and death [17, 19, 20, 38–41]. The difference between the PN and RN group will be indicated by an odds ratio and each event presented by count and percentage. The outcome will be measured over 2 years post-randomisation [17, 19, 20, 38–41].
- Progression to CKD stages 3, 4 and 5 (end stage renal failure) captured by eGFR. The difference between the PN and RN group will be indicated by an odds ratio and presented by the number of participants and percentage of each group who progress to the three CKD stages during the trial. The outcome will be measured over 2 years post-randomisation.
- Operative conversion to RN captured by conversion to RN. The outcome will only be observed in the PN group. It will be presented as the number and percentage of participants in the PN group whose surgery is converted to RN. The outcome will be measured before or during the index procedure.
- Patient acceptability of the intervention was captured by a theory-informed PQ (based on work by Sekhon et al. [42]) in both trial arms at 3 months post-intervention. Post-randomisation crossovers and preferences identified in screening logs will also be monitored during the trial to develop solutions to enhance recruitment as part of the embedded mixed methods trial process evaluation.

Long-term objectives:

- Participants can consent to potential future follow-up, through efficient means (such as routine data) as part of a separately funded study, allowing correlations of renal preservation with cardiovascular events, survival (cancer-specific, recurrence free and overall survival) and ESKD at 5–10 years.
- We aim to establish a well-characterised cohort of patients with RCC including clinical data, urine, blood and tumour specimens for future studies (based on separate funding).
- The core outcome sets (COS) for renal cancer [43] are currently being developed by other teams and due to complete within the lifetime of PARTIAL. Outcomes identified from the COS will be incorporated (where able) into PARTIAL data collection.

### Participant timeline {13}

**Fig. 1:**
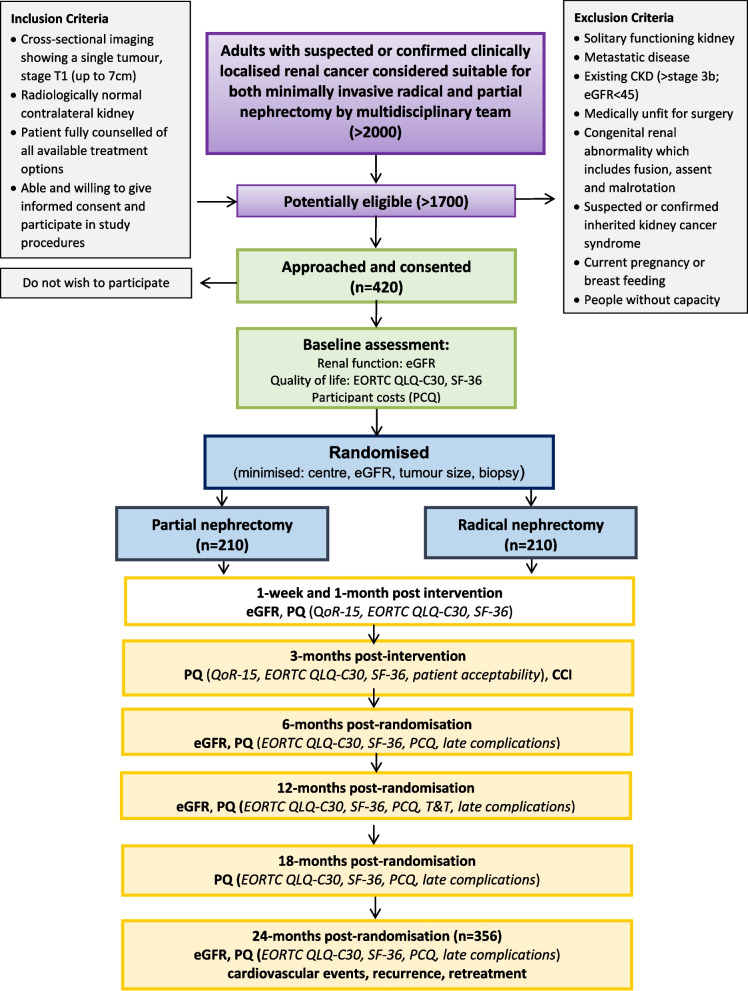
Participant timeline of the PARTIAL trial. CCI, Comprehensive Complications Index; CKD, chronic kidney disease; eGFR, estimated glomerular filtration rate; EORTC QLQ-C30, European Organisation for Research and Treatment of Cancer Quality of Life Questionnaire Core 30; SF-36, 36-Item Short Form Survey Instrument; PCQ, participant costs questionnaire; PQ, participant questionnaire; QoR-15, 15-Item quality of recovery scale; T&T, time and travel questionnaire

Figure 1 describes the participant timeline.

### Sample size {14}

Our sample size is based on the primary outcome of kidney function, and is also adequate for a 90% power non-inferiority test on the CCI. We will have precision to describe positive surgical margins and quality of life:

(i) For kidney function, we have used the width of the confidence interval between the groups in eGFR at 2 years: We estimate from systematic reviews that the absolute difference between PN and RN groups in arresting kidney function decline from baseline will be an eGFR of at least 10 ml/min/1.73m^2^, with a standard deviation of 16 [9, 19, 44]. This is a minimal clinically important difference informing international guideline recommendations for PN [45–48]. Based on the only randomised data available for PN vs RN, we predict a difference of at least 15 ml/min/1.73m^2^ at 2 years [3, 12]. As we require the confidence interval around the estimated difference to rule out a minimal clinically important difference of 10 ml/min/1.73m^2^, we have used a confidence interval of width 7. To be 90% sure our two-sided 95% confidence interval is at most 7, requires outcome data on 178 participants in each arm, or 356 participants in total. We will gain extra precision by using participants’ baseline eGFR in analysis.

We have inflated the sample size to account for 15% of potential attrition over 2 years to 420 randomised participants in total.

(ii) We require CCI outcome data on 380 procedures for 90% power to rule out a 4-point non-inferiority margin (i.e. we would tolerate up to 4 points higher CCI in the PN group) using the upper bound from a one-sided 97.5% confidence interval around the difference in means between the groups. The 4-point margin was derived using expert opinion and patient and public involvement on a non-inferiority margin for major complications, and from data on a minimally important difference in the CCI [32].
(iii) With 420 procedures, we will have adequate precision to describe rates of positive surgical margin over a range of scenarios. Positive surgical margin rates up to 5% may be expected, with only < 1% requiring re-treatment [49].
(iv) Our sample size also gives adequate power/precision to compare quality of life (expressed as area under curve analysis between randomisation and 24 months), with approximately 90% power to detect a third of standard deviation.

Based on Mayo Scoring System, rates of metastatic recurrence over 3 years for low-risk (most T1a/T1b) tumours are 2.1% [50]. In a contemporary series comparing PN with RN for T1b, 5-year metastases rates were 8.2% and 12.8%, respectively [51]. Nevertheless, survival rates for localised T1b renal cancer remain high with over 5-year cancer-specific survival rates for PN = 97.6% and RN = 95.5% [51]. Also, 2-year overall survival rates in the Van Poppell study were > 95% for both PN and RN [13]. Therefore, we do not anticipate any emerging patterns of differential outcome on the main measures of interest due to few events at 2 years.

For the process evaluation interviews, approximately 15 interviews per group will be conducted for participants who consented to the RCT and patients who did not consent to the RCT. A minimum of 15 clinical and recruitment staff at a minimum of 7 participating centres will also be interviewed. Sampling will be informed by Francis et al. [52]. All staff involved in discussing the PARTIAL trial with potential participants will be asked to routinely record consultations in which the trial is discussed.

### Recruitment {15}

Potential participants are identified following review by specialist MDT at UK hospitals. Potentially eligible patients may be identified in generalist urologist clinics in hospitals without specialist expertise in PN or RN. Such patients may be discussed at MDT meetings at specialist centres where their treatment may take place.

Local pathway and procedures at participating hospitals are different and the timing and mode of approach to eligible patients and the consent process may vary to accommodate both the local circumstances and the needs and preferences of the potential participant. A study poster may be displayed as a resource to support recruitment.

The embedded process evaluation aims to identify and address key challenges to trial recruitment and retention. It is modelled on the Quintet Recruitment Intervention [53] and augmented using a behavioural science approach to inform key components of data collection, analysis and development of feedback. The process evaluation includes the following: monitoring of participant flow at sites; audio-recording and analysis of trial consultations; and theory informed interviews with patients and site staff involved in the trial at a range of sites. The results of this evaluation are to be fed back to sites and included in ongoing site training events in real time to support and improve recruitment.

An embedded internal pilot phase with stop/go criteria will establish if recruitment is achievable.

## Assignment of interventions: allocation

### Sequence generation {16a}

Participants are randomly allocated using a remote central, computer-generated randomisation system accessed on the trial website (developed and administered by the Centre for Healthcare Randomised Trials (CHaRT), University of Aberdeen). The allocation algorithm uses centre, pre-operative eGFR (45–59; ≥ 60), tumour size (T1a; T1b), and pre-randomisation biopsy (yes; no) as minimisation covariates to allocate to PN or RN.

### Concealment mechanism {16b}

A remote, central, computer-generated randomisation system conceals the allocation sequence.

### Implementation {16c}

A delegated member of the local research team enrols the participant on the trial website. The remote, central, computer-generated randomisation system that is embedded on the trial website assigns the participant to either PN or RN.

## Assignment of interventions: blinding

### Who will be blinded {17a}

Participants and the local research team are informed which surgical intervention (PN or RN) has been allocated after randomisation. It is not possible to blind participants or the immediate clinical, nursing and research teams to the surgery they are receiving. However, both the kidney function and harms co-primary outcomes are determined objectively.

### Procedure for unblinding if needed {17b}

Not applicable—the trial is not blinded.

## Data collection and management

### Plans for assessment and collection of outcomes {18a}

Schedule of enrolment, interventions and assessments are summarised in Table 1.

**Table 1:** Schedule of enrolment, interventions, and assessments

	**Trial period**								
	**Enrolment & allocation**	**Post-allocation**							
		**Surgery**	**Post-surgery**			**Post-randomisation**			
**Timepoint**	**R0**	**S0**	**S0 + 1 week**	**S0 + 1 month**	**S0 + 3 months**	**R0 + 6 months**	**R0 + 12 months**	**R0 + 18 months**	**R0 + 24 months**
**Enrolment:**									
Eligibility screen	X								
Informed consent	X								
Randomisation	X								
**Interventions:**									
Minimally invasive partial nephrectomy		X							
Minimally invasive radical nephrectomy		X							
**Assessments:**									
Baseline characteristics	X								
Renal function: eGFR^a^	X		X	X		X	X		X
Quality of life: EORTC QLQ-C-30, SF-36	X		X	X	X	X	X	X	X
Participant cost questionnaire	X					X	X	X	X
Participant time and travel							X		
Comprehensive Complications Index					X				
Quality of recovery: QoR-15			X	X	X				
Late complications						X	X	X	X
Cardiovascular events									X
Recurrence^b^									X
Further treatment									X
Surgical details & resource use		X							
Pathology & positive surgical margins		X							
Patient acceptability					X				

*eGFR* estimated glomerular filtration rate, *EORTC QLQ-C30* European Organisation for Research and Treatment of Cancer Quality of Life Questionnaire Core 30, *QoR-15* 15-Item quality of recovery scale, *R0* date of randomization, *SF-36* 36-Item Short Form Survey Instrument, *S0* date of surgery

^a^There are preferable measurement windows for eGFR at key timepoints: 1 week post-surgery (within 1 week of surgery); 1 month post-surgery (± 1 week); 6 and 12 months post-randomisation (± 1 month); 24 month post-randomisation (± 3 months). All eGFR tests, including out of window observations, are recorded

^b^This outcome is not collected in participants who do not have confirmed renal cell carcinoma (RCC)

At baseline, the local research team captures baseline demographic and clinical information, including baseline renal function (eGFR) and tumour size and surgeon assigned location parameters (for PADUA classification [21]). Participants are asked to complete a baseline questionnaire prior to randomisation. During follow-up, the local research team collects outcomes from participant medical notes.

Renal function measurements (eGFR) are collected at key trial timepoints: at 1 week and 1 month post-intervention, and at 6, 12, and 24 months post-randomisation. The preferable window for the 1-week measurement is within 1 week of surgery. The preferable window for the 1-month measurement is ± 1 week. The preferable window for the 6- and 12-month measurements is ± 1 month. The preferable window for the 24-month measurements is ± 3 months. Where these key trial timepoints do not coincide with eGFR measurements taken as standard of care, the local research team requests that the eGFR measurement is taken either at a hospital outpatient clinic or in primary care. The results of all eGFR tests post-randomisation, up to 24 months post-intervention, including out of window observations, are recorded by the research team from laboratory records. Serum creatinine values are collected and will be used to calculate eGFR centrally for analysis.

PQs at 1 week, 1 month and 3 months post-intervention, and 6, 12, 18 and 24 months post-randomisation are administered by the trial office. Questionnaires are completed by the participant either electronically (with a link sent by email or text) or as a hardcopy (in the post with a reply-paid envelope provided), in accordance with their preference. Up to two reminders are sent to participants. The final reminder is by telephone. If there is no response by telephone, the final reminder is sent by post. The 1-week questionnaire is excluded from the final postal reminder due to potential overlap with the 1-month questionnaire.

For the process evaluation component, the number of participants screened, eligible, approached and randomised is recorded on clinic logs. All staff involved in discussing the trial with potential participants are asked to routinely audio-record consultations in which the trial is discussed. Semi-structured telephone interviews are conducted with participants who consented to the RCT, patients who did not consent to the RCT, and clinical and recruitment staff at participating centres.

### Plans to promote participant retention and complete follow-up {18b}

The process evaluation team will identify and address the key challenges to retention from the perspective of overall acceptability. Participant flow at sites, recruitment consultations, and interviews with patients and site staff will be analysed. Potential solutions in the form of action plans will be developed, implemented and evaluated on a rolling case basis.

Participants are sent up to two reminders to complete the follow-up questionnaires. First attempts and first reminders are emailed, posted or texted to participants, in accordance with their preference. The final reminder is attempted by telephone. If there is no response by telephone, the final reminder is sent by post. If questionnaires are returned as non-deliverable, attempts will be made by site staff or the trial office to trace the participant. Questionnaires are administered to all participants who were randomised in the trial, regardless of whether they had the surgery they were allocated, unless they have withdrawn from questionnaire follow-up.

Preferable measurement windows for the key eGFR measurements are specified within the protocol. An out of window measurement is preferable to no measurement at any timepoint. Therefore, all eGFR tests post-randomisation up to 24 months post-intervention, including out of window measurements, are collected and will be incorporated into the analysis.

All other outcomes are collected from the participant’s medical records, unless the participant has specifically withdrawn consent for collection of outcome data.

Participants are followed up for trial outcomes unless they have withdrawn from all aspects of the trial. All data collected up to the point of complete withdrawal from the trial is retained and used in the analysis. Participants for whom any outcome data are available are included in an intention to treat analysis.

### Data management {19}

Outcome data is either entered directly into the trial database (hosted by CHaRT, University of Aberdeen) or completed as a hardcopy and then entered onto the trial database by the local research team. Postal questionnaires are entered into the trial database by the trial office. The trial office works closely with local research teams to ensure the data is as complete and accurate as possible. For the first participants recruited at each site, the trial office reviews hardcopy case report forms and baseline participant questionnaires, entered by the local research team into the trial database, to check accuracy of data input. The trial database includes automated range and validation checks. Regular data accuracy spot checks are completed on questionnaires entered into the trial database by the trial office. All consent forms are checked for errors.

### Confidentiality {27}

Data collected during the course of the research is kept strictly confidential and accessed only by members of the trial team. Data may be looked at by individuals from the Sponsor organisation or NHS sites where it is relevant to the participant taking part in this trial. Personal data is retained only as long as is necessary for the purpose for which it is processed. The chief investigator and trial staff involved with this project will comply with the requirements of the General Data Protection Regulations (GDPR) and the Data Protection Act 2018. Trial staff based in Scotland will also adhere to the current version of the NHS Scotland Code of Practice on Protecting Patient Confidentiality. Access to collated participant data is restricted. All data input/access is via secure Virtual Private Network and/or secure website. Limited access measures, robust authentication and pseudonymisation are used to protect participant data. Published results will not contain any personal data that could allow identification of individual participants.

### Plans for collection, laboratory evaluation and storage of biological specimens for genetic or molecular analysis in this trial/future use {33}

We plan to seek separate funding to establish a well-characterised cohort of patient tissue including clinical data, urine, blood and tumour specimens which will allow future measures of biomarker for detection, surveillance and prognosis, and inform best treatment strategies.

## Statistical methods

### Statistical methods for primary and secondary outcomes {20a}

All statistical analyses will be pre-specified in a comprehensive Statistical Analysis Plan which will be agreed with trial steering committee (TSC) and data monitoring committee (DMC).

All analyses will be based on the intention-to-treat principle. The primary outcome, difference in kidney function decline, will be analysed using a mixed effects repeated measures generalised linear model that includes a random effect for participant and centre, with fixed effects for treatment, nominal time points, and design covariates and adjusted for baseline eGFR. Treatment effect estimates will come from the time-by-treatment interactions and the primary focus is on the 2-year timepoint. A second analysis of this outcome will use a latent growth model on kidney function measures at all time points to explore the trajectory of decline between groups. The CCI at 90 days will be compared between groups, initially using linear regression, using the bounds of the 95% CI around the difference, to test non-inferiority. However, there is potential for the CCI distribution to violate the assumptions of linear regression—if that occurs, we will use a more appropriate analysis method, e.g. beta regression on CCI scores transformed to the (0,1) interval. Positive surgical margins will be analysed as a binary outcome using logistic regression model including a random effect for centre, with fixed effects for treatment and design covariates. Treatment effects for positive surgical margins will be summarised using absolute percent differences.

The HRQoL outcomes will be analysed in two ways—an analysis of the area under curve and a repeated measures analysis using a time-by-treatment-interaction. Other secondary outcomes will be analysed in a similar way with generalised linear models appropriate for the distribution of the outcome. Treatment effects will be summarised with treatment estimates and 95% confidence intervals.

The trial includes a within trial and model based economic evaluation. Full details of the health economics analyses will be set out in the Health Economics Analysis Plan (HEAP).

The within trial economic evaluation will be conducted on an intention to treat principle. Results will be presented as a cost-consequence analysis and a cost-utility analysis from the NHS and personal social services perspective for the 24-month trial follow-up. A broader perspective incorporating costs to patients and their families will form part of sensitivity analysis. Our base case analysis will adopt standard purchasing approaches used in existing economic evaluations [54], but we will also explore alternative equipment costing assumption. The unit costs of NHS and personal social services resource use will be estimated from trial specific estimates and routine data sources [55, 56]. Unit costs will be combined with information on the use of services to estimate a cost for each participant and a mean cost for each intervention arm.

The relative changes in HRQoL captured by the SF-36 will be converted to SF-6D scores [57] and QALYs estimated using the area under the curve approach [58]. As part of sensitivity analysis, we will map the EORTC QLQ-C30 (administered at the same time points as the SF-36) on to the EQ-5D valuation set and these used to estimate QALYs.

An appropriate regression model will be fitted to estimate marginal costs and QALY gains, controlling for baseline covariates. Data will be presented as point estimates and bootstrapping techniques characterise imprecision [59]. The results will be presented as cost and QALY plots and as cost-effectiveness acceptability curves [60]. The cost-consequence analysis will be presented as a balance sheet to illustrate the trade-offs between different health outcomes.

An economic decision model (e.g. a microsimulation model) will extrapolate costs and outcomes over the lifetime of the patient. The model will be used to produce estimates of costs and QALYs. Cost-effectiveness will be reported as incremental cost per QALY gained at both 24 months and over the patient’s lifetime.

For both the within trial and the model-based analyses, costs and effects occurring after 1-year will be discounted at the recommended rates, currently 3.5% per annum.

### Interim analyses {21b}

There will be one analysis of effectiveness outcomes at the end of the trial after all follow-up is complete. There is no planned interim analysis for efficacy or futility. Safety data is monitored throughout the trial by an independent DMC.

### Methods for additional analyses (e.g. subgroup analyses) {20b}

Pre-planned subgroup analysis includes pre-operative eGFR, any comorbidity (diabetes affects 20%, hypertension affects 60% and cardiovascular disease affects 20%) [61], smoking status, age and size of tumour.

Pre-operative renal biopsy is not a standard of care across the UK and is not mandated for recruitment into the trial. Participants found to have a non-cancerous mass will remain in the trial. The effect of this will be explored by a post hoc sub-group analysis if the numbers in both confirmed RCC and no RCC are sufficient. If one of the groups is too small for a sub-group analysis, a sensitivity analysis will be undertaken.

For the economic evaluation, sub-group and sensitivity analyses will be replicated where these are relevant to the estimation of costs and QALYs. Deterministic sensitivity analyses will be combined with the trial-based stochastic or model-based probabilistic analyses to explore other forms of uncertainty (e.g. different cost assumption, inclusion of participant costs, QALYs based on EORTC QLQ-C30 responses).

For the process evaluation, analysis of patient recruitment pathways using the Screened, Eligible, Approached, Randomised (SEAR) framework will be applied to identify and assess areas of complexity and protocol compliance [53]. The transcripts of the recruitment consultations will be analysed using content and thematic analysis, focusing on modifiable aspects of recruitment consultations. A novel mixed-methods approach combining consultation timings (time spent explaining aspects of the RCT) and qualitative interpretation of the conversation (quanti-qualitative appointment timing) may be used for the purpose of analysis as appropriate. Sample characteristic information will be presented using frequencies. Data collection and analysis of patient and site staff interviews will be informed by the Theoretical Domains Framework and/or the Theoretical Framework of Acceptability, which has been applied in existing studies exploring trial feasibility in other contexts [62].

### Methods in analysis to handle protocol non-adherence and any statistical methods to handle missing data {20c}

The sensitivities of all treatment effect estimates to missing outcome data will be explored. Patterns of missing data will be described, and multiple imputation (under missing at random assumption) and pattern mixture models will be used.

### Plans to give access to the full protocol, participant level-data and statistical code {31c}

The full protocol is available on the NIHR website under award listing NIHR133561 [63]. Data availability is detailed in the ‘Availability of data and materials’ section of this publication.

## Oversight and monitoring

### Composition of the coordinating centre and trial steering committee {5d}

The trial office is based in the Centre for Healthcare Randomised Trials (CHaRT), Aberdeen Centre for Evaluation, University of Aberdeen. The trial manager in CHaRT takes responsibility for the day-to-day trial activities and the data co-ordinator provides clerical support. The experienced qualitative/mixed methods researcher takes responsibility for the process evaluation component of the trial. The trial office team meet at least monthly during the trial.

The Project Management Group (PMG), consisting of grant holders and representatives from the trial office, supervises the trial. The PMG meet approximately every 3 months and has clinical, surgical and methodological expertise.

The TSC, with independent members, oversees the conduct and progress of the trial. The TSC Charter documents the terms of reference of the TSC and is filed in the Trial Master File.

### Composition of the data monitoring committee, its role and reporting structure {21a}

An independent DMC oversees the safety of subjects in the trial and comprises clinical and statistical experts. The DMC Charter documents the terms of reference of the DMC and is filed in the Trial Master File. The DMC meet at least annually during the trial and make recommendations to the TSC.

### Adverse event reporting and harms {22}

In PARTIAL, serious adverse events (SAEs) are recorded between commencement of surgical treatment (entering the anaesthesia suite) until the participant exits from the trial (24 months after randomisation or withdraws from collection of data). The investigator queries relevant SAEs at every visit and within follow-up questionnaires. SAEs must be serious and related to the research procedures required by the protocol. SAEs do not include procedures administered as standard of care, complications of surgery, prolongation of hospitalisation without an associated adverse event, additional medication required above that normally expected, emergency presentations and admissions, routine admissions for pre-planned events, or any adverse event that would be captured as a secondary outcome for the trial. Complications and secondary outcomes are captured in case report forms and PQs. The investigator (or medically qualified delegate) reviews medical documentation related to the event, completes the SAE form and reports to the trial office within 24 h of knowledge of the event. If a potential SAE is recorded on a PQ, the trial office will seek further information from the investigator team. The investigator, medically qualified delegate or chief investigator assess the seriousness, relatedness and expectedness of the event. The trial office notifies unexpected SAEs to the sponsor within 24 h of receipt of a signed SAE notification. The chief investigator or delegate notifies unexpected SAEs to the research ethics committee within 15 days of the chief investigator becoming aware of the event. The trial oversight committees and funder review all SAEs in regular progress reports.

### Frequency and plans for auditing trial conduct {23}

The trial is audited and monitored by the sponsor. A sponsor-approved trial monitoring plan is in place which details oversight arrangements, training and the monitoring of data collection, participant consent and safety by the trial office. Sites may have further monitoring arrangements locally.

### Plans for communicating important protocol amendments to relevant parties (e.g. trial participants, ethical committees) {25}

Any amendment to the protocol or other approved documents will be reviewed by sponsor (and funder where appropriate) before application to the Research Ethics Committee. Exceptionally, if urgent safety measures are required, then the sponsor is notified as soon as possible. Sites are notified in accordance with current guidelines. The trial registry is updated accordingly. Participants are notified if the amendment impacts on agreed participant activities.

## Dissemination plans {31a}

The main trial findings will be published, and a plain English summary will be sent to participants. Trial findings will also be disseminated to professionals involved in the trial, including general practitioners (GPs) of participants, investigators at sites and site staff. Detailed plans for dissemination will be considered and developed with input from patient and public involvement partners through the duration of the trial. Dissemination plans will be finalised as part of the close-out plans.

## Discussion

Patient treatment preference is a known issue in surgical trials [64], impacting recruitment into the trial. In our focus groups, patients with lived experience of PN or RN (*n* = 24) described diverse experiences in being counselled about the surgical options. However, the vast majority understood that there was a choice between PN and RN for smaller tumours and agreed that a clinical trial is urgently required to determine which treatment is superior in terms of relative benefits and harms for intermediate sized tumours. In PARTIAL, only patients that are deemed by the MDT to be suitable for both procedures will be approached for participation and patients will be counselled about both PN and RN as part of routine clinical practice. We have embedded an evaluation of the recruitment process to identify and address any potential challenges to recruitment and randomisation in real time. This package of activities is modelled on established methods that have successfully explored and addressed randomisation acceptability, by both potential participants and surgeons, in several surgical trials [64, 65]. The findings will be developed into pragmatic solutions and directly fed into the recruitment processes to improve trial delivery across sites.

We learned from our patient focus groups that surgeon preference was a major determinate in patient decision-making, which could impact recruitment into the trial. National data revealed a spread of treatment approaches for T1 tumours, in keeping with uncertainty around best practice [25]. There is a growing narrative in the published field about feasibility of PN for large T2 tumours [66], although this is not reflected in UK practice where only 3% were undertaken in 2019 [25], most likely in complex scenarios. Our eligible patient population is therefore limited to T1 tumours. We explored surgeon preference in our scoping exercise with National BAUS Section of Oncology urologists (*n* = 110), presenting several potential scenarios based on tumour morphological parameters (the main determinant in having a preference between PN and RN). For small (T1a) peripheral tumours, there was a strong preference for PN (93%). However, 78% of urologists explicitly stated a willingness to randomise T1b tumours (with the solitary exception of hilar endophytic T1b cases) and endophytic hilar T1a tumours; this therefore defines our expected trial population. We have not restricted our inclusion criteria by tumour morphology as nephrotomy scoring systems (e.g. PADUA, RENAL) are not used in routine clinical practice. Our inclusion criteria instead enable recruitment of patients with localised T1 tumours where there is equipoise within the MDT.

We considered biopsy-proven RCC as an inclusion criterion and specifically explored this in our national urologist questionnaire and patient focus groups. Although biopsy is routinely offered in selected cases by 70% of clinicians, it is not routinely taken up by patients who often elect to proceed direct to surgery having expressed anxiety about waiting for biopsy and delaying treatment. Therefore, we have not mandated biopsy as an inclusion criterion. Where biopsy is part of local standard of care, approximately 10% may have non-conclusive biopsy. For these patients, if the surgeon and patient are in equipoise in relation to full or partial nephrectomy, they are eligible for the trial and can be randomised. If, following treatment, it becomes apparent that there was no RCC present, the participant will remain in this pragmatic, intention to treat trial. All outcomes (apart from those secondary outcomes specifically related to RCC, including local recurrence, need for re-treatment) are relevant and will be collected from this group.

PARTIAL is the first RCT comparing minimally invasive RN with minimally invasive PN for patients with localised T1 RCC. The current gaps in evidence of the benefits of partial nephrectomy for intermediate sized T1b tumours and small complex T1a tumours, increased surgical complications and excision of more tissue (reducing preservation of renal function) make the potential gains over radical nephrectomy less clear. The health economic benefits are also uncertain. The outcomes of PARTIAL will have significant relevance to improving patient outcomes, cost-effectiveness for the NHS and providing clinical guidance based on high-quality evidence. PARTIAL could lead to significant changes in how early-stage kidney cancer is managed, with the potential to reduce the need for more extensive surgeries and associated long-term renal complications.

Recruitment began 2 months later than anticipated on 01 March 2023, with the approval of the funder. Site set-up progressed on target but recruitment is slower than expected. The funder approved an extension to the internal pilot study with revised targets. The revised internal pilot was extended 12 months to run to 31 November 2024. On completion of the revised internal pilot, we requested an overall extension to the trial with updated targets and milestones. A multipronged approach to improve participant recruitment has been developed following the process evaluation, which we intend to publish separately.

## Trial status

The current protocol is version 3, 01 August 2024. The first participant was recruited into the trial on 18 April 2023. Recruitment was initially expected to complete 31 December 2024 with last participant follow-up expected in December 2026. The funder is supportive of an overall extension to the trial with a revised recruitment end date of 30 September 2026 and last participant follow-up expected in September 2028.

## Supplementary Information

Additional file 1.

Additional file 2.

Additional file 3.

## Data Availability

The final trial dataset will be accessible to the trial statistician and health economist for the purpose of trial analysis. The final trial dataset may also be made available on reasonable request. A request to access the final trial datasets generated during the trial should be directed in the first instance to the chief investigator (Professor Naeem Soomro; n.soomro@nhs.net; and copied to datasharing@abdn.ac.uk). The datasets collected in questionnaires at all timepoints, transcripts of process evaluation interviews, and the baseline, surgery, discharge and pathology, 3 month (CCI), eGFR follow-up, 24 month and serious adverse event case report forms for all participants recruited to the trial will be available. The dataset will be available in fully anonymised electronic form, at an individual level and in accordance with participant consent. The data dictionaries, trial protocol, statistical analysis plan, health economics plan, patient information leaflet and template case report forms will be available on request to facilitate interpretation of data. Questionnaire templates, or parts thereof, may be available pending review of the relevant licensing agreements. Electronic data for the trial will be available within a local repository at the University of Aberdeen and will be retained for a period of at least 5 years after close of trial in accordance with funder, sponsor and local archiving procedures. Applicants will require to complete a data request form that will be reviewed by a Data Sharing Committee, which includes the chief investigator. Applications will be considered on a case-by-case basis from bonafide researchers. We are obligated to ensure that optimal use is made of the data that are collected for research and we recognise the value of sharing individual-level data. The interests of research participants, researchers and other stakeholders will be considered when considering each application. A fully authorised data sharing agreement will be required prior to the release of data.

## Abbreviations

CCI: Comprehensive Complications Index
CHaRT: Centre for Healthcare Randomised Trials
CKD: Chronic kidney disease
CKD-EPI: Chronic Kidney Disease Epidemiology Collaboration
DMC: Data monitoring committee
eGFR: Estimated glomerular filtration rate
EORTC QLQ-C30: European Organisation for Research and Treatment of Cancer Quality of Life Questionnaire Core 30
ESKD: End stage kidney disease
GCP: Good Clinical Practice
GP: General practitioner
HRQoL: Health-related quality of life
MDT: Multidisciplinary team
PADUA: Preoperative Aspects and Dimensions Used for Anatomic classification
PCQ: Participant costs questionnaire
PQ: Participant questionnaire
PMG: Project management group
PN: Partial nephrectomy
QALY: Quality adjusted life year
QoR-15: 15-Item quality of recovery scale
RCC: Renal cell carcinoma
RCT: Randomised controlled trial
RENAL: Radius Exophytic/endophytic Nearness Anterior/posterior Location nephrometry score
RN: Radical nephrectomy
SAE: Serious adverse event
SF-36: 36-Item Short Form Survey Instrument
T&T: Time and travel questionnaire
TSC: Trial steering committee
