# Supplementary material for: PARTIAL: study protocol for a clinical and cost-effectiveness of complex PARTIAL vs radical nephrectomy for clinically localised renal cell carcinoma randomised trial
Source: Trials. 2026 Mar 20;27:331. doi: 10.1186/s13063-026-09624-4 (PMC13126806; doi:10.1186/s13063-026-09624-4)
Supplement: Supplementary file 1 — Additional file 1. [file 13063_2026_9624_MOESM1_ESM.pdf]

<<site logo>>

TRIAL CONSENT FORM
PARTIAL

Participant Trial Number

Please INITIAL all boxes

By INITIALLING each box and signing this form:

1) I confirm that I have

read and understood the Participant Information Leaflet about the PARTIAL trial (Version number X, date XX/XX/XX). I have had the opportunity to consider the information, ask questions and have had these answered satisfactorily. ☐

2) I understand that

a) my participation is voluntary and that I am free to withdraw at any time without giving any reason, without my medical care or legal rights being affected. Data collected up until the point of withdrawal may still be used in analysis ☐

b) I will be asked to complete questionnaires detailed in the Participant Information Leaflet ☐

c) relevant sections of my medical notes and data collected during the trial may be looked at by individuals directly involved in the trial, from the Newcastle upon Tyne Hospitals NHS Foundation Trust or from the NHS Boards or Trusts, where it is relevant to my taking part in this research. I give permission for these individuals to have access to them ☐

d) relevant data collected for the purpose of the trial together with personal contact details will be confidentially and securely stored on computer servers maintained by the University of Aberdeen in accordance with the Data Protection Act. I agree that the trial co-ordinators can use my contact details to send me trial questionnaires and to contact me by phone or post or email or text ☐

e) the information collected about me may be shared anonymously with other researchers to support future research ☐

f) I agree to my General Practitioner being informed of my participation in this trial ☐

I agree to take part in the PARTIAL trial ☐

OPTIONAL

I understand that I may be contacted in the future for long-term follow-up ☐

I am willing to be contacted in the future about participating in other relevant ethically approved research. ☐

Your signature (participant)	Name in BLOCK capitals	Date

To be completed by the local team member taking consent

I confirm that I have explained to the person named above, the nature and purpose of the trial and the procedures involved.

Signature	Name in BLOCK capitals	Date
