# Supplementary material for: PARTIAL: study protocol for a clinical and cost-effectiveness of complex PARTIAL vs radical nephrectomy for clinically localised renal cell carcinoma randomised trial
Source: Trials. 2026 Mar 20;27:331. doi: 10.1186/s13063-026-09624-4 (PMC13126806; doi:10.1186/s13063-026-09624-4)
Supplement: Supplementary file 2 — Additional file 2. [file 13063_2026_9624_MOESM2_ESM.pdf]

PARTIAL

The PARTIAL study

A randomised trial of the clinical and cost effectiveness of partial vs radical nephrectomy for clinically localised renal cell carcinoma

Please help us to find out which type of surgery is better for long-term function of the kidney for patients with an early stage tumour on their kidney

Participant Information Leaflet

PARTIAL study website: w3.abdn.ac.uk/hsru/PARTIAL

Invitation to take part

We would like to invite you to take part in the PARTIAL study, a research study looking at the long-term impact on function of the kidney between two types of surgery for kidney cancer. The study is funded by the National Institute of Health and Care Research (NIHR, the research arm of the NHS).

Before you decide if you would like to take part, it is important for you to understand why the research is being done and what it will involve.

The first part of this Participant Information Leaflet tells you the purpose of the study and what will happen to you if you take part.

Then, in the second part, we give you more detailed information about how the study is run.

Please take time to read the information carefully, which has been written with the help of patient representatives who have had surgery on their kidney in the past. Talk to others about the study if you wish. Ask us if there is anything that is not clear or if you would like more information.

You can find contact details for the PARTIAL study team at the back of this leaflet.

What is the purpose of this research study?

Most people are born with two kidneys, but people can manage with one working kidney. Our kidneys help us remove waste products from the body. We can measure how well a person's kidneys are working using a test called estimated Glomerular Filtration Rate (eGFR for short, and sometimes called a kidney function test).

Every year, over 13,000 people in UK have a tumour on their kidney: some of these are a kidney cancer, others turn out not be a cancer. Often, these tumours are identified when they are at the early stages of growth (up to 7cm), and if there is no spread to other parts of the body there are two main types of surgery that can be done:

- Removal of the whole kidney if there is a normal kidney on the other side (known as total nephrectomy)
- Removal of part of the kidney (where the tumour is) (known as partial nephrectomy).

Both surgeries are routinely used in the NHS, and both are good options to treat early-stage kidney cancer. Both surgeries have potential benefits and risks and there is no good evidence for doctors and surgeons about which surgery is better for long-term benefits. You can read more about the benefits and risks of each type of surgery on page 4 of this information leaflet.

It is also unclear which type of surgery is the best option for patients. The way we find this out is to conduct what is called a randomised trial. We do randomised trials when there is more than one treatment option available for patients with a disease and we don't know which treatment is best. In this situation, people are randomly allocated to different treatment groups. The results from the different treatment groups are compared to see if one treatment is better. To try to make sure the groups are the same, each patient is randomly allocated to one of the two treatment groups. This process is essential to avoid bias: such as if the groups receiving each treatment are the same, any differences in the results can only be down to the treatments. Therefore, randomisation means that the results are more reliable.

The PARTIAL study will compare the two different types of surgery mentioned above. To compare them, we will need to collect information on a number of things, including any complications of surgery, kidney function, and quality of life for two years after surgery. The results of the PARTIAL study will help doctors, surgeons, patients and health services decision-makers understand whether it is better to remove the whole kidney or part of the kidney.

The study will run for 5 years and aims to recruit around 420 participants from hospitals across the UK which offer both types of surgery.

What would taking part involve?

If you decide to take part in this research study, you will be randomly allocated to one of two surgical treatments (total nephrectomy or partial nephrectomy). Being randomly allocated means that neither you nor your doctor will be able to decide which type of surgery you receive. You will have an equal chance of getting either of the surgeries. We will tell you which group you have been allocated to before you have your surgery.

Either

- You will be in the group where the whole kidney is removed (total nephrectomy)

Or

- You will be in the group where part of the kidney is removed (partial nephrectomy)

If you decide to take part, you should keep a copy of this leaflet and we will ask you to complete a consent form confirming that you are happy to take part. You can complete this consent form during a routine visit to the hospital, or we can post it to you to do at home, or you can complete it on computer, tablet or mobile phone. We will then ask you to complete a questionnaire (which should take less than 20 minutes to complete) about your quality of life and your recent contact with the NHS.

The surgical team will arrange a date for your surgery. After you have had your surgery:

- we will collect some information from your medical notes about the surgery that you had.
- you will receive all the usual care within the NHS. If you need any further treatment, your GP or hospital team will arrange this for you.
- as part of the PARTIAL study, we will follow you up for up to 24 months. The table below summarises what will happen while you are part of this research study

	Complete a questionnaire	Have a blood test to check your kidney function
When you join the study	✔	
While you are in hospital after your surgery		✔
1 week after surgery	✔	
1 month after surgery	✔	✔
3 months after surgery	✔	
6 months after you join the study	✔	✔
12 months after you join the study	✔	✔
18 months after you join the study	✔	
24 months after you join the study	✔	✔

We will ask you to complete questionnaires about your quality of life and your recovery from surgery at 1 week, 1 month and 3 months after you have had surgery. Each of these questionnaires will take about 20 minutes to complete.

We will then ask you to complete questionnaires at 6, 12, 18 and 24 months after you join the study. Each of these questionnaires will ask about your quality of life, any complications you may have had after your surgery, and about your contacts with the NHS. Each of these questionnaires will take about 20 minutes to complete. At the 12 month timepoint we will ask you a few more questions about your contacts with the NHS, for example how far it is to travel to your GP surgery or hospital, and how much it costs you to attend these appointments. These extra questions will take about 10 minutes to complete.

We can send each of these questionnaires to you by post, along with a pre-paid envelope for you to return them to the PARTIAL study office in Aberdeen, or we can send you an email or text message (depending on your preference) with a link to complete the questionnaire online. We may send you reminders about completing the questionnaires to make sure relevant information about you is recorded for the study.

After your surgery, as part of standard NHS care, you will have regular blood tests to check your kidney function. We will collect the results of these tests from your medical records. If for any reason, you do not receive these kidney function tests as part of standard NHS care, we will invite you to have these tests done – the first one will be done while you are in hospital after your surgery. We will then invite you to have a test at 1 month after your surgery, and then at 6, 12 and 24 months after you join the study. These might be done at the hospital or at your GP practice.

After 2 years we will also collect information from your medical notes about anything else significant that has happened to you since you joined the study.

We will also ask a small number of people to take part in telephone or in-person interviews to find out how they make decisions about taking part (or not) in the PARTIAL study. We will ask you whether you would like to know more about these interviews and give you another information leaflet to read if you are interested.

What are the possible benefits of taking part?

You may not benefit personally from taking part in the study. There is evidence that people who take part in clinical studies may have better outcomes. By taking part, you will be directly helping us to inform the treatment of future patients who need to have kidney surgery.

What are the possible disadvantages, risks and side effects when taking part?

We do not think that there are any possible disadvantages to you. If you take part in the PARTIAL study, there should be no **additional** risk to you. Both types of surgery are already being used in the NHS to treat patients who need kidney surgery and your surgeon will confirm that you are suitable to have both types of surgery before you take part.

There are risks associated with all surgical procedures. Steps are always taken to ensure that these risks are minimised, whether in routine hospital care or clinical research. Whichever treatment group you are allocated to, your surgery will be performed by a competent and trained surgeon. As part of your routine care, your surgeon will tell you about the potential risks and benefits of each type of surgery. Some of the main risks and benefits are described below.

**Total nephrectomy surgery where the whole kidney is removed**	**Partial nephrectomy surgery where part of the kidney is removed**
• This can be an easier surgery for the surgeon to do.	• It can be more difficult for the surgeon to remove just part of the kidney. Sometimes during this type of surgery, the surgeon has to take out the whole kidney.
• There is a lower chance of bleeding and urine leakage from the surgery.	• There is a higher chance of bleeding and urine leakage from the surgery
• If there is a cancer in the kidney, there is a higher chance of it all being removed.	• If there is a cancer in the kidney, there is a small risk of some cancer being left behind which in most cases does not require additional treatment.

• If the whole kidney is removed, the overall kidney function may be worse because only one kidney is left to do the work of removing waste products from the body.<br><br>• The chance of developing kidney disease is higher	• If part of the kidney is left in place, the overall kidney function may be better than removing the entire kidney because the part that is left behind can still help get rid of waste products from the body.<br><br>• The chance of developing kidney disease is lower

Do I have to take part?

No. It is entirely up to you whether or not you take part. Please take as much time as you need to make this decision. You can read this information leaflet as many times as you wish and ask your surgeon or doctor and/or research nurse as many questions as you like. There is also 'PARTIAL Decision Support Information' available to help patients like you make informed decisions about taking part in this study. If you have not received a copy, please ask your research team or the Study Office. Contact details can be found on the back of this leaflet.

If you decide not to take part, your surgeon will discuss with you which type of surgery you will have.

You can decide at any time to withdraw from the study. This decision will not affect the standard of care you are receiving now or in the future. If you make this decision, you should continue attending appointments with your consultant and/or GP as part of your routine care.

If you decide to withdraw from this research study, we will keep and continue to use all your previously collected data. We would like to continue collecting information about your health from NHS records, your hospital and your GP. If you do not want this to happen, tell us and we will stop. This information will remain confidential and will not be used for any other purpose. To safeguard your rights, we will use the minimum personally-identifiable information possible.

We need to manage your records in specific ways for the research to be reliable. This means that we won't be able to let you see or change the data we hold about you.

What happens when the research study stops?

If the study is stopped earlier than expected for any reason, we will tell you and arrange continuing care for you.

What if relevant new information becomes available?

Sometimes during the course of a research study, new information becomes available about the treatment that is being studied. If this happens, the PARTIAL Study Office staff will contact you to let you know about the choices available to you. However, we are not aware that any new, relevant information is likely to become available before the end of this study.

What if there is a problem?

If you have a question or concern about the study, you can ask to speak with the research team who will do their best to answer your questions. Contact details for your local study

nurse and the Study Office can be found on the last page of this information sheet. If you wish to complain formally or have any concerns about any aspects of the way you have been approached or treated during this study, you can do this through the normal NHS Complaints Procedure. Details can be obtained from your hospital.

We do not expect any harm to come to you by taking part in this study. In the event that something does go wrong, and you believe that you are harmed by taking part in this study, you have the right to pursue a complaint and seek compensation through the research sponsors of this study, Newcastle upon Tyne Hospitals NHS Foundation Trust:

Tel: [redacted]
Email: [redacted]
Address: Patient Relations Department, The Newcastle upon Tyne Hospitals NHS Foundation Trust, The Freeman Hospital, Newcastle upon Tyne, NE7 7DN.

If you are harmed due to someone's negligence, then as a patient of the NHS, you may have grounds for legal action. You may have to pay for your legal costs yourself.

Will my taking part in the study be kept confidential?

Yes. We will follow ethical and legal practice and all information which is collected about you for the purpose of this research study will be handled in strict confidence and securely stored by the University of Aberdeen.

Who will have access to my information if I take part in the study?

We will use information from you and your medical records for this research project. This information will include your name, contact details, date of birth and NHS number (England, Wales and Northern Ireland only) or CHI number (Scotland only). People will use this information to do the research or to check your records to make sure that the research is being done properly. People who do not need to know who you are will not be able to see your name or contact details. Your data will have a code number instead. We will keep all information about you safe and secure. Once we have finished the study, we will keep some of the data so we can check the results. We will write our reports in a way that no-one can work out that you took part in the study.

The local research team at the hospital that you were recruited in and where you have the surgery will have access to your information. They will use your name and contact details to contact you about the study, to make sure that relevant information about the study is recorded for your care, and to oversee the quality of the study. They may contact your GP to request routine blood tests to check your kidney function and to collect relevant information from your medical records.

The study team, who are based in the Centre for Healthcare Randomised Trials (CHaRT) at the University of Aberdeen, will have access to your information to contact you about the study, for example to send you the questionnaires, and for quality control purposes, such as auditing the data collection process. All electronic data collected for the purpose of the research study will be confidentially and securely stored on computer servers maintained by the University of Aberdeen. The local research team will pass information collected from you and your medical records to the study team.

We will tell your GP you are taking part.

The statistical analysis of this study is being conducted at the University of Aberdeen. The health economic analysis of this study is being conducted at The London School of Hygiene & Tropical Medicine. To maintain confidentiality, these teams will only analyse completely anonymous data. (Anonymous data does not include names or addresses, and it is not possible to identify individual participants from anonymous data).

Other individuals from the Newcastle upon Tyne Hospitals NHS Foundation Trust (who sponsor the study), and the Research and Development Department of your local NHS Organisation may look at your medical records and data collected for the study, to check that the study is being carried out correctly and to check the accuracy of the research study. All will have a duty of confidentiality to you as a research participant.

Other researchers may wish to access anonymous data from this study for future research. If this is the case, they would be expected to follow legal, data protection and ethical guidelines. It will not be possible to identify you from this data. The information will only be used for the purpose of health and care research and cannot be used to contact you or affect your care.

Your rights to access, change or move your information are limited, as we need to manage your information in specific ways in order for the research to be reliable and accurate.

How long will my information be kept?

All information which is collected about you during the research, including identifiable data, will be held securely for 5 years after the study has finished in accordance with Sponsor requirements and data legislation.

Who is responsible for my information?

The Newcastle upon Tyne Hospitals NHS Foundation Trust is the data controller for this study and is responsible for looking after your information, using it properly and complying with your rights.

You can find out more about how we use your information:

- at www.hra.nhs.uk/information-about-patients/
- in our leaflet available from https://www.newcastle-hospitals.nhs.uk/help/privacy/privacy-notice-for-patients/
- by asking one of the research team
- by sending an email to the Sponsor Data Protection Officer at [redacted]
- by ringing the Newcastle upon Tyne Hospital Data Protection Officer on [redacted]

What will happen to the results of the study?

We will use the results of the study to make recommendations on the best type of surgery for people with a growth on their kidney. We will publish the results of this study in scientific journals and present the information at appropriate meetings. You will not be identified in any publication resulting from the study. We will let you know the results of the study when it is finished unless you tell us that you do not wish to know.

Who is organising and funding the study?

This study is sponsored by Newcastle upon Tyne Hospitals NHS Foundation Trust who have overall responsibility for the management of the study. The study is funded by the National Institute of Health and Care Research (NIHR, the research arm of the NHS). The research is being carried out by a group of experienced kidney doctors and surgeons from across the UK, in collaboration with researchers from the Centre for Healthcare Randomised Trials (CHaRT), a UKCRC registered Clinical Trials Unit at the University of Aberdeen and The London School of Hygiene & Tropical Medicine.

Who has reviewed the study?

All research in the NHS is looked at by an independent group of people, called a Research Ethics Committee (REC), to protect your interests. This study has been reviewed and given a favourable opinion by REC South West – Central Bristol.

Thank you for reading this

Thank you for taking the time to read this information leaflet. We hope that it has been helpful in enabling you to decide if you would like to participate in the PARTIAL study. Please ask us if you have questions or would like more information about the study.

Further information and contact details

If you have any questions or would like any more information, please contact:

PARTIAL Study Office<br>Centre for Healthcare Randomised Trials (CHaRT),<br>Health Services Research Unit<br>University of Aberdeen<br>Health Sciences Building<br>Foresterhill<br>Aberdeen AB25 2ZD<br>Tel: [redacted]<br>Email: [redacted]<br>Web: **w3.abdn.ac.uk/hsru/PARTIAL**	**<<Local centre contact details>>**

The Chief Investigators for the study are Professor Naeem Soomro and Professor Rakesh Heer. You can contact them through the PARTIAL study office (details above).

Kidney Cancer UK and Kidney Cancer Scotland are helping to support the PARTIAL study. The charity provides information, support and advice to people with kidney cancer, carers and their families. Find out more at https://www.kcuk.org.uk/ or by contacting their support line [redacted]
