# Supplementary material for: PARTIAL: study protocol for a clinical and cost-effectiveness of complex PARTIAL vs radical nephrectomy for clinically localised renal cell carcinoma randomised trial
Source: Trials. 2026 Mar 20;27:331. doi: 10.1186/s13063-026-09624-4 (PMC13126806; doi:10.1186/s13063-026-09624-4)
Supplement: Supplementary file 3 — Additional file 3. [file 13063_2026_9624_MOESM3_ESM.pdf]

PARTIAL Decision-Support Information

The clinical study is known as PARTIAL, and compares two different surgeries:

- Removal of the whole kidney (known as total nephrectomy)
- Removal of part of the kidney (known as partial nephrectomy)

The PARTIAL study aims to find out which type of surgery is better for long-term function of the kidney for patients with an early stage tumour on their kidney. You are invited because you are suitable to participate in the study and receive either of the surgeries. Your surgeon will have reviewed your case and considered you to be suitable for both surgical treatments. If you decide to take part, you will be randomly allocated to one of two surgical treatments (total or partial nephrectomy). You will have an equal chance of getting either of the surgeries.

Instructions on how to use the decision support information

- Please read this decision-support information alongside or after you have read the participant information leaflet. These documents aim to help patients like you make informed decisions about taking part in the PARTIAL study.
- Indicate the extent to which each reason (to choose or avoid the PARTIAL study) matters to you **using numbers (1 to 5) in the table below**. There is a space at the bottom of the table to add any "other reasons to choose the PARTIAL study" and "other reasons to avoid the PARTIAL study".
- Please review the information below and discuss it with your surgeon and with others, if you wish, including family or friends.

Will the PARTIAL study suit me?

"How much it matters": - Use numbers (1 to 5) to indicate (1 = if it does not matter at all and 5 = if it matters a lot)

Possible reasons to choose the PARTIAL study	How much it matters	Possible reasons to avoid the PARTIAL study	How much it matters
**Randomisation**<br>Some people may think it is advantageous to have their surgery chosen randomly when there is no good evidence for the doctors and surgeons about which is best over the long term.		**Randomisation**<br>You will be allocated to total or partial nephrectomy at random. There will be a 50:50 chance that you will receive either of the two surgeries. Randomisation means that neither you, your surgeon, nor the healthcare team will decide which surgery you will receive. Once randomisation is done we will tell you which surgery you will receive before you have your surgery.	
**Helping future patients**<br>By taking part, you will be directly helping future patients who need kidney surgery.		**Additional tasks**<br>If you decide to take part, you will have to complete questionnaires that you would not normally complete. You may also have to have additional blood tests.	
**Closer monitoring**<br>Your progress will be monitored carefully over the next two years. You will likely receive more attention from your healthcare team and careful monitoring of your condition and the possible side effects of surgery. After two years, you will be followed up longer as part of your routine care.		**Preferences**<br>You may have a preference for which type of surgery you want. However, this should be carefully explored as there is no good evidence of which surgery is better for you.	
Other reasons to **choose** the PARTIAL study		Other reasons to **avoid** the PARTIAL study	

PARTIAL
Decision-Support Information

Kidney tumour: Treatment options

The possible side effects and your risk of getting them are shown below. These side effects can vary a lot from patient to patient; please ask your surgeon about these and their impact on you as an individual. Evidence of long-term side effects is unclear due to a lack of research in the area. These side effects and risks are related to having a nephrectomy, not specifically to taking part in this study.

Side effect	Risk following total nephrectomy (where the whole kidney is removed)	Risk following partial nephrectomy (where part of the kidney is removed)
**Bleeding**<br>Requiring blood transfusion (i.e., a process of transferring donated blood) or conversion to open surgery	**Possibly lower chance**<br>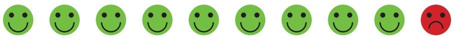<br>1 in 10 patients<br>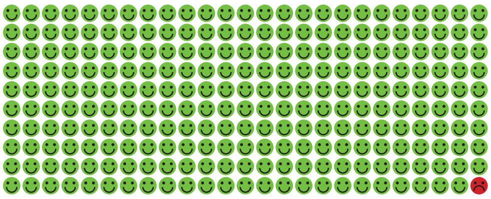<br>1 in 250 patients	**Possibly higher chance**<br>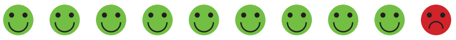<br>1 in 10 patients<br>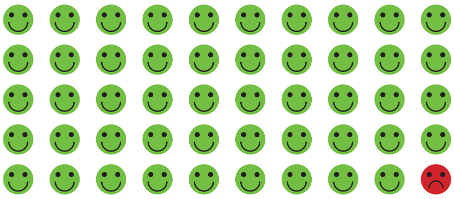<br>1 in 50 patients
**Injury**<br>Recognised (or unrecognised) injury to organs/blood vessels requiring conversion to open surgery (or another surgery in the future)	**Possibly higher chance**<br>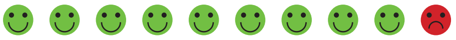<br>1 in 10 patients<br>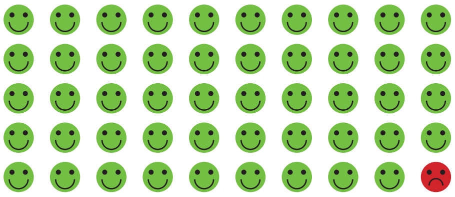<br>1 in 50 patients	**Possibly lower chance**<br>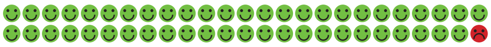<br>1 in 50 patients<br>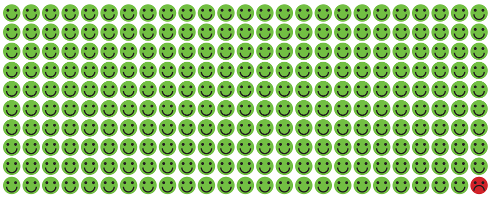<br>1 in 250 patients
**Recovery**	Most people return to work or normal activities after four to six weeks.	Most people return to work or normal activities after four to six weeks.
**Removal of all cancer**	If there is a cancer in the kidney, there is a higher chance of it all being removed.	If there is a cancer in the kidney, there is a small risk of some cancer being left behind, and very few patients will require any additional treatment.
**Recurrence of the cancer**	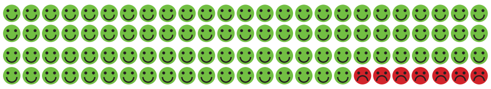<br>7 in 100	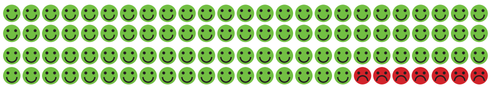<br>7 in 100
**Survival**	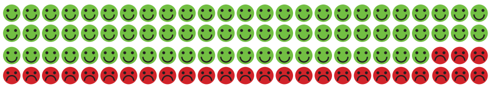<br>72 in 100 will live for 10 years or more.	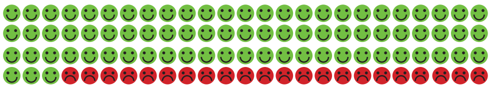<br>78 in 100 will live for 10 years or more.
